# Sensory nerve conduction stimulus threshold measurements of the infraorbital nerve and its applicability as a diagnostic tool in horses with trigeminal-mediated headshaking

**DOI:** 10.1186/s12917-024-04068-x

**Published:** 2024-05-16

**Authors:** Jasmin Nicole Nessler, Julien Delarocque, Tanja Kloock, Lara Twele, Stephan Neudeck, Nina Meyerhoff, Franziska Riese, Jessica-M. V. Cavalleri, Andrea Tipold, Karsten Feige, Tobias Niebuhr

**Affiliations:** 1 Department of Small Animal Internal Medicine and Surgery, University of Veterinary Medicine Hannover, Bünteweg 9, 30559 Foundation, Hannover, Germany; 2 Clinic for Horses, University of Veterinary Medicine Hannover, Bünteweg 9, 30559 Foundation, Hannover, Germany; 3 Clinical unit of equine internal medicine, Department of small animals and horses, University of veterinary medicine, Vienna, Austria

**Keywords:** Trigeminal-mediated head shaking, Sensory nerve conduction stimulus threshold, Infraorbital nerve, Neuropathy

## Abstract

**Background:**

To determine whether sensory nerve conduction stimulus threshold measurements of the infraorbital nerve are able to differentiate horses with idiopathic trigeminal-mediated headshaking (i-TMHS) from healthy horses and from horses with secondary trigeminal-mediated headshaking (s-TMHS). In a prospective trial, headshaking horses were examined using a standardized diagnostic protocol, including advanced diagnostics such as computed tomography and 3-Tesla-magnetic resonance imaging (MRI), to differentiate s-TMHS from i-TMHS. Clinically healthy horses served as controls. Within this process, patients underwent general anesthesia, and the minimal sensory nerve conduction stimulus threshold (SNCT) of the infraorbital nerve was measured using a bipolar concentric needle electrode. Sensory nerve action potentials (SNAP) were assessed in 2.5–5 mA intervals. Minimal SNCT as well as additional measurements were calculated.

**Results:**

In 60 horses, SNAP could be recorded, of which 43 horses had i-TMHS, six had suspected s-TMHS, three horses had non-facial headshaking, and eight healthy horses served as controls. Controls had a minimal SNCT ≥ 15 mA, whereas 14/43 horses with i-TMHS and 2/6 horses with s-TMHS showed a minimal SNCT ≤ 10 mA. Minimal SNCT ≤ 10 mA showed 100% specificity to distinguish TMHS from controls, but the sensitivity was only 41%.

**Conclusion:**

A minimal SNCT of the infraorbital nerve ≤ 10 mA was able to differentiate healthy horses from horses with TMHS. Nevertheless, a higher minimal SNCT did not exclude i-TMHS or s-TMHS and minimal SNCT does not distinguish s-TMHS from i-TMHS.

**Supplementary Information:**

The online version contains supplementary material available at 10.1186/s12917-024-04068-x.

## Introduction

Headshaking is a syndrome characterized by involuntary flicking or jerking movements of the head. Clinical signs can be present at exercise, rest, or both. Often, clinical signs are accompanied by additional signs of nasal irritation, such as nose rubbing, sneezing or striking the nose [1–3]. The severity of clinical signs varies between individuals and ranges from very mildly affected horses with occasional clinical signs, to severely affected horses, which are unsafe to ride or handle [1]. Clinical signs are believed to be a form of neuropathic facial pain syndrome [4, 5]. In some horses, a great variety of discomforting, inflammatory, or painful diseases can cause headshaking. Contrary to that, in horses with idiopathic headshaking, the trigeminal nerve is suggested to be primarily affected. In those horses, no associated structural pathologic lesion can be found with a complete diagnostic work-up [1, 6, 7]. The diagnostic work-up in horses with headshaking is often challenging, as the current approach involves excluding structural diseases that could cause pain or discomfort and consequently secondarily result in headshaking [1, 6]. Minor findings often prompt questions regarding their clinical significance. Excluding structural headshaking is particularly crucial, as addressing underlying diseases may allow for more targeted treatment. Therefore, the development of a biomarker for diagnosing idiopathic headshaking is desirable.

Recently, somatosensory evoked potentials and nerve conduction stimulation threshold measurements were performed in six horses with idiopathic headshaking along several locations of the maxillary nerve. In these horses, the nerve conduction stimulus threshold was significantly decreased on both sides compared to controls [8]. Interestingly, other features, such as nerve conduction velocity or amplitude of the evoked potentials, were unaltered compared to controls [8]. The authors therefore concluded that, in horses with idiopathic headshaking, the stimulation threshold is decreased, probably caused by a functional rather than a structural impairment of the trigeminal nerve [8, 9]. Additionally, upon histopathological examinations of the trigeminal nerves of horses with headshaking, no pathologic abnormalities can be detected [8, 10]. Based on these results, idiopathic headshaking is termed trigeminal-mediated headshaking (TMHS) in recent literature [5, 10–13].

The aim of this prospective study was to establish a feasible protocol to measure the nerve conduction stimulus threshold of the infraorbital nerve bilaterally in horses for diagnostic purposes and compare the results to healthy controls. The authors hypothesize that in idiopathic trigeminal-mediated headshaking (i-TMHS) the conduction threshold would be as low as that in horses with underlying pathologies involving the trigeminal nerve (= secondary trigeminal-mediated headshaking, s-TMHS). Furthermore, the association of nerve conduction threshold with the severity of clinical signs, the practicability of this method, side effects, and the ability to differentiate s-TMHS from i-TMHS was investigated.

## Materials and methods

**Fig. 1:**
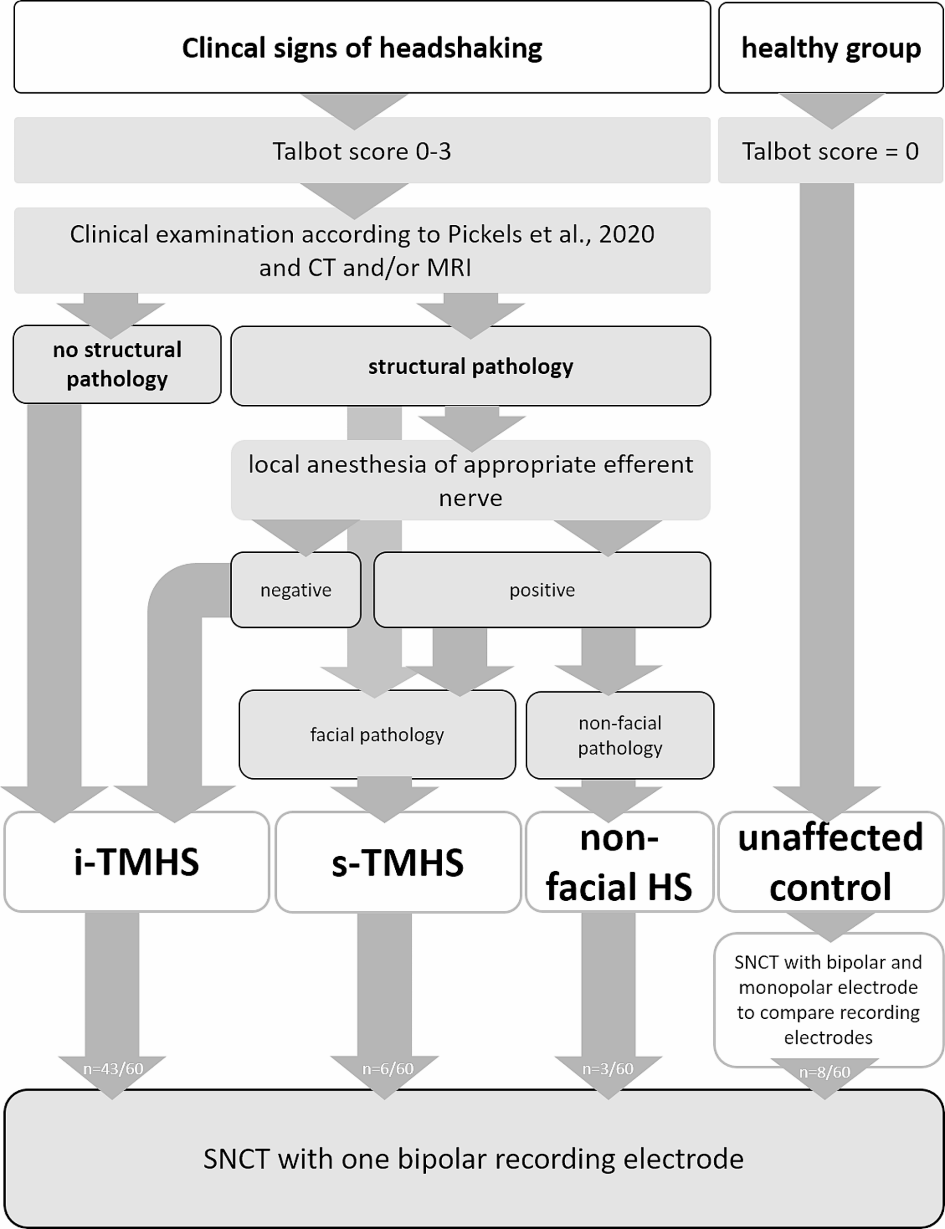
Workflow to diagnose the underlying cause of headshaking (HS) in horses. i-TMHS = idiopathic trigeminal-mediated headshaking; s-TMHS = secondary trigeminal-mediated headshaking; CT = computed tomography; MRI = magnetic resonance imaging; SNCT = sensory nerve conduction stimulus threshold, n = number of horses

In this monocentric prospective study, client owned horses with clinically confirmed signs of headshaking were included. All examinations were performed with the informed owner’s written consent and according to the university’s guidelines. A standardized diagnostic work up for headshaking patients was used (Fig. 1). For the examination of the control horses, animal experiment permission according to the Review Board for the Care of Animal Subjects of the district government (Lower Saxony, Germany) and federal law was granted (33.14-42502-04-13/1235).

The study population consisted of 15 Hanoverians, ten Westphalian, nine Warmbloods, six Oldenburg, four Ponies, three Thoroughbred, each two Trakehner, Icelandic horses, and Haflinger, each one Fresian, Irish Sporthorse, Knabstrupper, Quarter Horse, Tinker; in 2 horses the breed was not documented. With a median age of 9.45 years (range 2–26 years), the study population consisted of 19 mares, 4 stallions, 35 geldings and two horses of undocumented sex.

### Diagnostic work-up

The standardized diagnostic work-up of horses with headshaking included a thorough patient history, a daily clinical examination including determination of clinical sign severity, and evaluation of hematology and blood chemistry. The signs were scored according to the score published by Talbot et al. [14]. In this scoring system, a score of 0/3 reflects no clinical signs at the time of examination whereas a score of 3/3 describes severe signs, including behavioral changes and refusal to move [14]. Furthermore, all horses underwent endoscopic, radiographic, dental, ophthalmic, neurologic, and orthopedic examinations. Subsequently, all horses underwent general anesthesia for computed tomography (CT- BrillianceTM CT – Big Bore Oncology Scanner, Philips Medical Systems, Best, The Netherlands), ear endoscopy and if possible magnetic resonance imaging of the head (MRI, 3.0 T scanner, Achieva, Philips Medical Systems, Best, The Netherlands). If an approachable pathologic lesion was found and suspected to be the cause of the clinical signs, nerve blocks of the affected area were performed with an appropriate local anesthetic if possible. If amelioration of clinical signs was evident afterwards and the lesion was within the innervation region of the trigeminal nerve, the horse was considered to have s-TMHS. If, due to an unapproachable location of the lesion within the innervation region of the trigeminal nerve, no local anesthesia was performed, and the lesion was estimated to be significant, then the horse was also considered affected by s-TMHS. If the lesion was in a region not innervated by the trigeminal nerve, the horse was considered to be affected by non-facial headshaking (non-facial HS), e.g. neck pain. If the complete diagnostic work up failed to uncover any pathology or if clinical signs persisted after local anesthesia, the horse was diagnosed with i-TMHS (Fig. 1).

In several horses, local anesthesia of the maxillary nerve was performed as described previously [7], and clinical amelioration of clinical signs was documented.

### Anesthesia

For diagnostic work up and for sensory nerve conduction measurements in general anesthesia, the horses were premedicated with xylazine to effect (0.6–0.8 mg/kg IV) (Xylavet, CP-Pharma, Germany). Induction of anesthesia was performed by guaifenesin 50 mg/kg IV (My50, CP-Pharma, Germany) followed by thiopentone 6 mg/kg IV (Thiopental Inresa, Inresa, Germany). Horses were kept in anesthesia stage III plane 2–4 [15]. In case of insufficient anesthetic depth, ketamine 1 mg/kg IV (Narketan, Vetoquinol, Germany) was added to the protocol. Following orotracheal intubation the animals were connected to an anesthesia machine (Vet.-Tec. Model LAVC 2000, J.D. Medical Distributing Company, AZ, USA) via a circle breathing system. Anesthesia was maintained by isoflurane (Isofluran CP, CP-Pharma, Germany) in 100% oxygen and xylazine to effect (0.4–0.8 mg/kg/h). The horses were mechanically ventilated with a positive inspiratory pressure (PIP) of 20–25 cmH2O. The PIP and the respiratory rate were adjusted accordingly to maintain an end-tidal carbon dioxide (PE´CO2) of 45 ± 5 mmHg. Lactated Ringer’s solution (Ringer-Laktat-Lösung, B. Braun Melsungen, Germany) and dobutamine (Dobutamin-ratiopharm, Ratiopharm, Germany) were infused to effect to maintain the mean arterial pressure (MAP) ≥ 60 mmHg. Anesthetic monitoring included sidestream capnography, a lead II electrocardiogram, invasive arterial blood pressure measurement, arterial blood gas sampling and the measurement of inspiratory and end-expiratory gas concentrations. At the end of the procedure the horses received flunixin-meglumine 1.1 mg/kg IV (Flunidol RPS, CP-Pharma, Germany) and were placed in a padded recovery box. Postanesthetic sedation was performed by xylazine 0.1 mg/kg IV and all horses recovered from general anesthesia by head-and-tail rope assistance.

### Sensory nerve conduction study

**Fig. 2:**
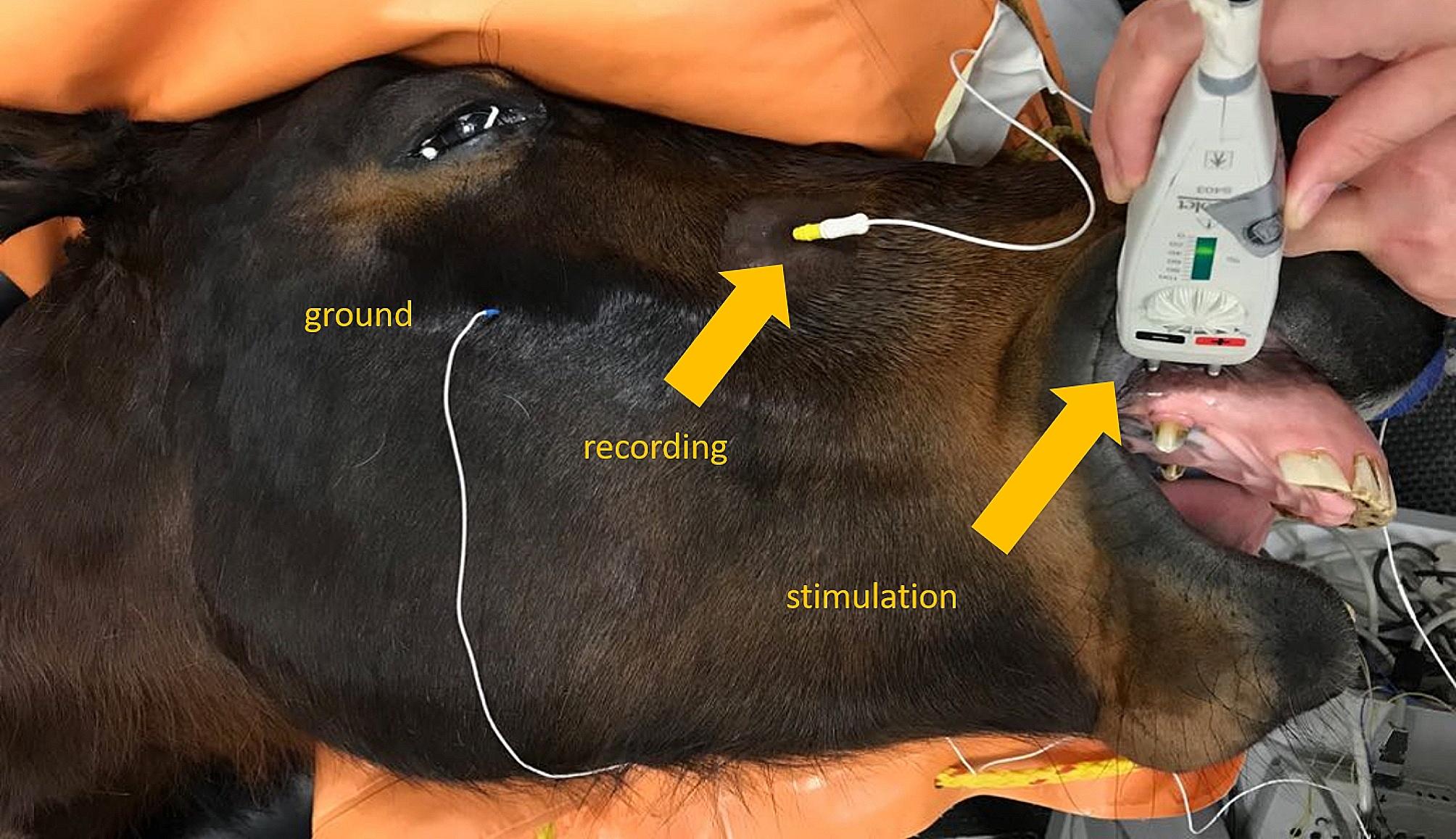
Sensory nerve action potential measurements: electrode placement, recording with concentric bipolar needle electrode

After a one-week recovery period, a sensory nerve conduction study of the infraorbital nerve was performed in all patients. The study was performed under general anesthesia following the protocol of Aleman et al. 2013 [8, 9] but, instead of two monopolar needle-electrodes, a single bipolar concentric needle was used for recording (s. below: pretest). After aseptic preparation of the underlying skin, the bipolar concentric needle electrode for recording was placed in close proximity to the infraorbital nerve at the infraorbital foramen, with the bevel directed toward the nerve (Fig. 2).

Furthermore, the study protocol of Aleman et al. 2013 [8, 9] was shortened to reduce the risk of general anesthesia. Therefore, only measurements of the infraorbital nerve were performed at the level of the infraorbital foramen. Each recording was the average of at least 250 individual stimulations. Stimulus intensity was reduced stepwise starting at 25 mA. Bilaterally, sensory nerve conduction velocity (SNCV), highest amplitude of sensory nerve action potential (SNAP) either from the right or left infraorbital nerve, and minimal stimulus intensity required to generate a visible SNAP either from the right or left infraorbital nerve were documented. The individual sensory nerve conduction stimulus threshold (SNCT) was considered the lowest stimulus intensity needed to detect a SNAP in each patient. If a different stimulation threshold was detected between the left and right infraorbital nerves of an individual, the lower threshold value was used for further evaluation and considered the minimal SNCT. SNCV for each horse is given as the mean from the left and right infraorbital nerves.

### Pretest to compare recording needle electrodes

To assure that the changed selection of needle electrodes will not alter threshold measurements, recordings with two monopolar needle-electrodes were compared to recordings with a single bipolar concentric needle electrode in five healthy control horses. The first measurement was performed with two monopolar recording electrodes placed as described previously for the measurement of the infraorbital nerve [8]. Subsequently, monopoplar electrodes were removed and measurements were repeated with one concentric bipolar electrode as described above in each horse. Minimal SNCT were compared for both electrodes.

### Statistical analysis

For statistical analysis, SAS Enterprise Guide ® 7.1 (SAS Institute Inc., NC, USA) and GraphPad PRISM 10.0.0 (GraphPad Software, Inc., La Jolla, CA USA) were used. The test for Gaussian distribution was performed with the Kolmogorov–Smirnov normality test and further statistical tests were chosen accordingly. If group size was small, non-parametric tests were chosen. Comparison of minimal SNCT with bipolar and monopolar electrodes was performed with a paired Mann Whitney test. An unpaired t-test was used to compare the mean SCNT of horses with a Talbot score of 0 to horses with scores of 1 to 3. Minimal SNCT, depending on the Talbot score or type of headshaking (i-TMHS, s-TMHS, non-facial and unaffected), was analyzed with Kruskal-Wallis test followed by Dunn’s multiple comparison’s test. Finally, Talbot scores were compared across types of headshaking using the Kruskal-Wallis test, followed by Dunn’s test. *P* values of *p* < 0.05 were considered significant. Sensitivity, specificity, and negative and positive predictive values were calculated using a 2 × 2 table.

## Results

### Pre-test: comparison of monopolar versus bipolar concentric needle recordings in unaffected horses

Recordings with two monopolar needle-electrodes were compared to recordings with a single bipolar concentric needle in healthy control horses to show that there is no significant difference between the methodology of Aleman et al. [8, 9] and our study. The recordings conducted with one bipolar concentric needle and with two monopolar needles were compared in 5 healthy horses. The SNCT of both infraorbital nerves was recordable with a bipolar concentric needle in 3/5 horses and only unilaterally in the other 2/5 horses. In contrast, with two monopolar recording needles, SNCT was only recordable unilaterally in 4/5 horses and in 1/5 horses no SNCT was obtained. Failure to record was most often caused by muscle artifacts or 60 Hz artifacts or no waveform could be elicited at all, independent of the needle’s location. If a SNAP was detected, the minimal SNCT did not differ significantly between recordings with monopolar or bipolar needle electrodes (bipolar concentric: *n* = 5, mean 18 mA [15–20 mA]; monopolar: *n* = 4, mean 16.25 mA [15–20 mA]; *p* = 0.171 (Supplemental Table 1); therefor, a bipolar concentric needle was used for all further analysis.

### Sensory nerve conduction studies in horses with and without signs of headshaking

Initially, 53 horses with signs of headshaking and 8 healthy control horses were recruited. In one horse with headshaking, electrodiagnostic measurements could not be performed due to technical issues. This horse was excluded from the study and was not considered for further analysis. Sensory nerve conduction measurements were performed in total in 60 horses. In six horses a SNAP was only recorded unilaterally due to technical problems. However in 54 horses SNAP were recorded on both sides (Supplemental Table 2).

Of the total of 60 horses with clinical signs of headshaking (52 headshaking and 8 controls), six horses were diagnosed with s-TMHS, and three horses were diagnosed with non-facial headshaking, as in those two groups, clinical relevant structural disease were found within the diagnostic process for each patient, which were likely to cause pain or discomfort and therefor cause clinical signs of headshaking. In the other headshaking horses (*n* = 43) no pathologic lesion was detected during the diagnostic work up or, if a potential structural cause was identified, local anesthesia of the corresponding efferent nerve did not show amelioration of headshaking (*n* = 15); therefor, i-TMHS was diagnosed.

In 19 horses, local anesthesia was performed, of which two distinct areas were anesthetized in three horses. Of 19 horses, 9 horses had structural lesions that were considered significant and might cause clinical signs of headshaking. The anesthetized regions were as follows: *n* = 15 maxillary nerve, *n* = 5 insertion point of the nuchal ligament at the occipital bone, *n* = 1 thoracic epiaxial muscles, and *n* = 1 temporomandibular joint. In several horses more than one side was anesthetized. In four horses, local anesthesia led to clear amelioration of signs of headshaking. Of those four horses, two were considered to show non-facial headshaking and two s-TMHS. The horses with i-THMS (*n* = 15/19) showed no amelioration of clinical signs of headshaking after local anesthesia, of which 12 horses received local anesthesia of the maxillary nerve.

Findings in s-TMHS horses were equine odontoclastic tooth resorption and hypercementosis (EOTRH), otitis media, bacterial sinusitis, temporohyoid osteoarthropathy, bilateral idiopathic levator nasolabialis myopathy, mandibular condylar cyst, and severe compression of the maxillary nerve by a dental root. Findings in non-facial headshaking horses were severe insertional desmopathy of the nuchal ligament, cervical arthrosis, back pain, and cholesteatoma. More than one disease was diagnosed in one horse in each group (Supplemental Table 2).

The SNCV was calculated in 50 horses. The mean SNCV was 73.6 m/s (56–90 m/s) in i-TMHS, 74.8 m/s (61.5–100 m/s) in s-TMHS, 72.3 m/s (69–79 m/s) in horses with non-facial headshaking, and 58.7 m/s (35–92.5 m/s) in unaffected horses (*p* > 0.24).

The mean highest SNAP amplitude was 57.36 µV (8-135 µV) in i-TMHS, 57.00 µV (25–135 µV) in s-TMHS, 61.33 µV (29–68 µV) in horses with non-facial headshaking, and 48.50 µV (10–124 µV) in unaffected horses (*p* > 0.333).

**Fig. 3:**
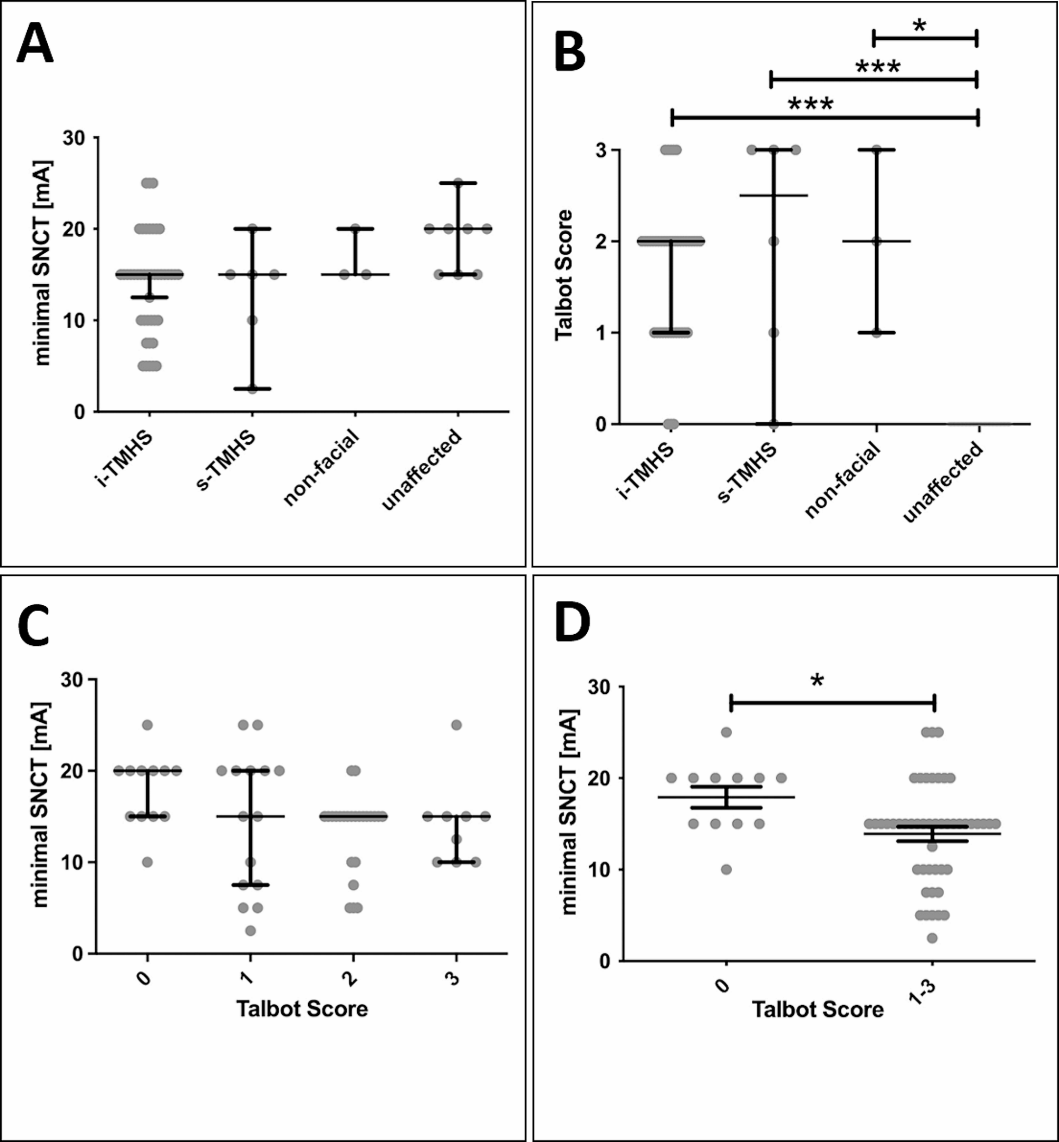
Minimal sensory nerve conduction stimulus threshold (SNCT) of the infraorbital nerve and clinical Talbot score. Horses with idiopathic trigeminal-mediated headshaking (i-TMHS) showed lower minimal SNCT than unaffected horses **(A)**. Unaffected horses showed significantly fewer headshaking clinical signs (Talbot Score) than horses with i-TMHS, s-TMHS or non-facial headshaking **(B)**. The Talbot score was not associated with minimal SNCT in affected and unaffected horses **(C)**, but minimal SNCT was significantly different in horses without clinical signs of headshaking (Talbot 0) compared to horses with signs of headshaking (Talbot ≥ 1). mA = milliamps. Bars present the median ± 95% confidence interval (in A-C) and mean ± SD (in D). ***p < 0.001

**Fig. 4:**
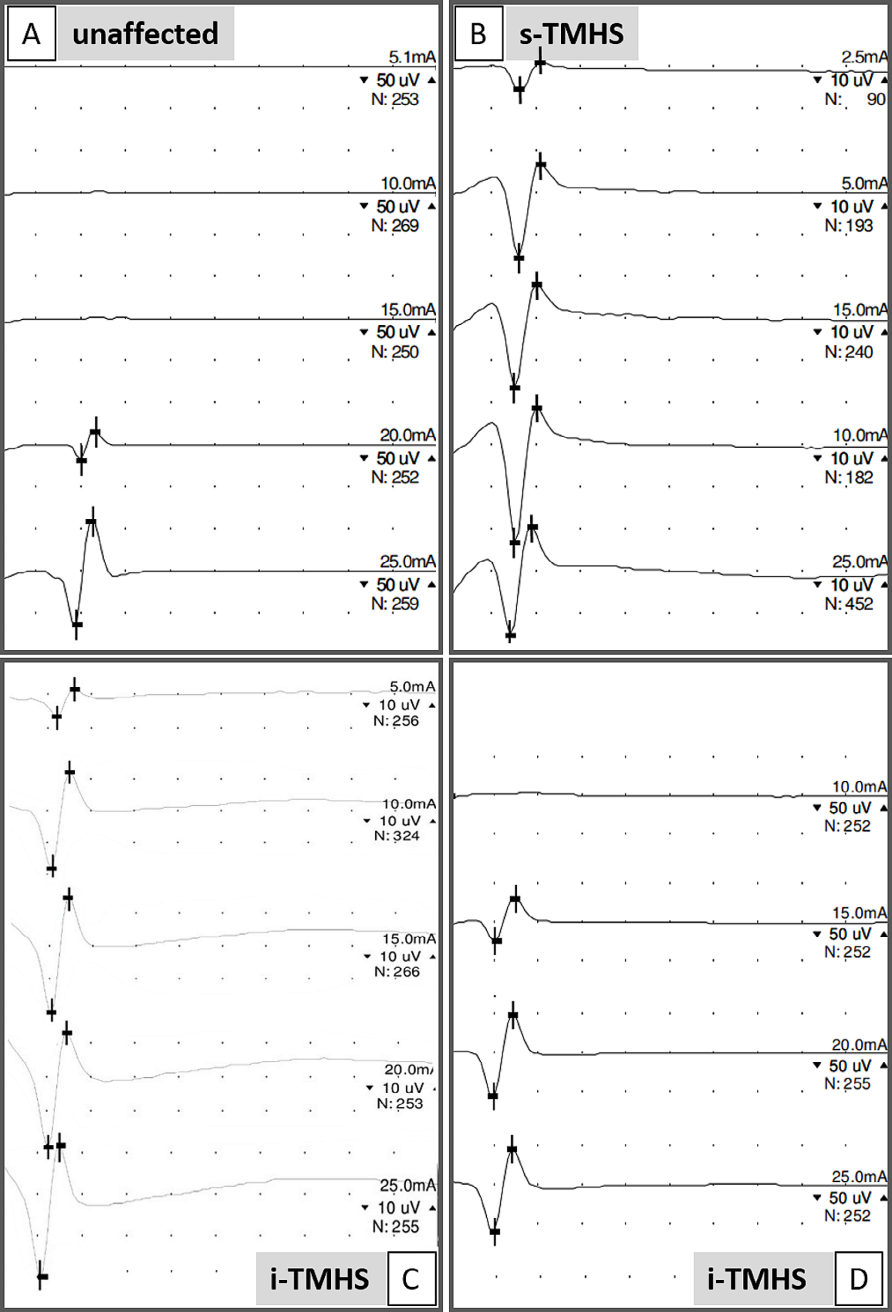
Sensory nerve conduction stimulus threshold (SNCT) measurements exemplary in a horse without clinical signs of headshaking (control, A: SNCT = 20 mA) and in horses with idiopathic trigeminal-mediated headshaking (i-TMHS, C: SNCT = 5 mA, D: SNCT = 15 mA) or secondary trigeminal-mediated headshaking (s-TMHS, B: SNCT = 2.5 mA). Each waveform represents the average of several repetitive sensory nerve action potentials; the number of repetitive stimulation are given as N. Measurements were performed in decreasing stimulus intensity, starting with 25 mA, until no waveform can be elicited anymore (bottom to top). Stimulation intensity is given in milliamps (mA). Space between horizontal marks show amplitude, and vertical marks show peak latencies. Please note the different scales for the individual horses displayed in micro Volts (µV)

All unaffected horses had a minimal SNCT ≥ 15 mA while the mean minimal SNCT was 18.75 mA (15–25 mA). Of the horses with i-TMHS, 14/43 had a minimal SNCT of ≤ 10 mA. In horses with i-TMHS mean minimal SNCT was 14.07 mA (5–25 mA). Of the horses with s-TMHS, 2/6 horses had a minimal SNCT ≤ 10 mA and mean minimal SNCT was 12.92 mA (2.5–20 mA). Of these two horses, one had severe EOTRH in the maxillary incisor teeth and the other was diagnosed with a mandibular condylar cyst. None of the horses with non-facial headshaking had SNCT ≤ 10 mA and mean minimal SNCT was 16.67 mA (15–25 mA). Due to the low number of horses with non-facial headshaking, they were not considered for further statistical analysis of minimal SNCT. There was a no significant difference in minimal SNCT between groups (*p* > 0.078) (Fig. 3). Examples of SNCT studies are given in Fig. 4.

The specificity of SNCT ≤ 10 mA to distinguish between unaffected horses and horses with i-TMHS was 100%. Nevertheless, the sensitivity and negative predictive values were rather low (Table 1).

**Table 1:** Sensitivity, specificity, negative (NPV) and positive predictive value (PPV) of minimal sensory nerve conduction stimulus threshold (SNCT) ≤ 10 mA to distinguish between diseases. i-TMHS = idiopathic trigeminal-mediated headshaking; s-TMHS = secondary trigeminal-mediated headshaking; unaffected = healthy control horses

	i-TMHS (*n* = 43) versus unaffected (*n* = 8)	TMHS (= i-TMHS + s-TMHS, *n* = 49) versus unaffected (*n* = 8)	i-TMHS (*n* = 43) versus s-TMHS (*n* = 6)
**Sensitivity**	32.6%	41.0%	32.6%
**Specificity**	100.0%	100.0%	66.7%
**PPV**	100.0%	100.0%	87.5%
**NPV**	21.6%	11.5%	12.1%

The Talbot scoring of clinical signs was 0/3 in the healthy controls, 0–3/3 in horses with i-TMHS, 0–3/3 in horses with s-TMHS, and 1–3/3 in horses with non-facial headshaking (*p* < 0.0001; Fig. 3). The minimal SNCT differed significantly between horses with a Talbot score of 0 (mean 17.92 mA (10–25 mA) and horses with a Talbot score ≥ 1 (mean 13.91 mA (2.5–25 mA); *p* = 0.0137). Three horses with i-TMHS and one with s-TMHS showed no clinical signs during the time of examination (Talbot score 0/3) and of those, only one horse had a minimal SNCT ≤ 10 mA while the other three had minimal SNCT ≥ 15 mA.

SNCT measurements of the infraorbital nerves bilaterally took less than 20 min after the examiners gained some practice (approximately after five horses). Side effects consisted of mild hematoma formation at the recording site in 2/60 horses. No exacerbation of headshaking clinical signs after SNCT measurements was observed by either the clinicians or reported by the owners.

## Discussion

Idiopathic trigeminal-mediated headshaking is associated with distress in affected horses and owners and reduces the quality of life of both [1]. Although it is a common disorder, its etiology and pathophysiology are largely unknown and therefore, a simple procedure to diagnose this disease is still lacking. To date, the diagnosis of i-TMHS is based on the exclusion of underlying structural diseases causing facial neuropathic discomfort or pain [2]. Aleman et al. were able to show that the trigeminal nerve function was altered in i-TMHS by detecting decreased SNCT of the trigeminal nerve at several locations [8]. Therefore, the present study aimed to develop a quick and feasible protocol to measure SNCT and to evaluate its capability to distinguish between horses with i-TMHS, s-TMHS, and healthy horses in a clinical setting.

In this study, SNCT measurements were easy and fast to perform -apart from general anesthesia, which is essential to prevent artificial electromyographic movement artifacts [16]- with only minimal side effects, consisting of mild hematoma formation at the electrode insertion site.

The results confirm in a larger population that horses suffering from headshaking can have a lower SNCT compared to healthy controls, as published by Aleman et al. [8], but it cannot differentiate between i-TMHS and s-TMHS. Nevertheless, it seems, that decreased minimal SNCT of the infraorbital nerve does not occur in horses with clinical signs of headshaking due to non-facial pain.

To measure sensory nerve action potentials, a previously published and slightly adapted method [8] was used to make it a feasible diagnostic tool in daily practice. One of the alterations was using one bipolar concentric needle for recording instead of two monopolar needles. Bipolar concentric recording needle electrodes produced a more reliable recording with fewer artifacts, were easy to place and showed comparable results of SNCT compared to two monopolar recording electrodes. Nevertheless, it must be considered that, due to this change, SNAP amplitudes might be lower with bipolar concentric needles as the recording diameter is generally lower than in recordings with monopolar needles [16]. Subsequently, this might influence the recorded minimal SNCT. To exclude the impact of different recording electrodes, we compared recording with two monopolar needle electrodes to recordings with one concentric bipolar electrode and found no significant difference in minimal SNCT. However, recording with two monopolar electrodes produced more artifacts and SNAPs were less reliably recorded. Therefore, we proceeded using a concentric bipolar recording needle electrode.

Aleman et al. found, that the minimal SNCT that differentiates between healthy horses and horses with headshaking was ≥ 10 mA for healthy horses [8], while in our study the minimal SNCT was ≥ 15 mA for healthy horses. Therefore, we suggest that hospital specific reference values must be established before this method is used as recommended for other electrophysiological tests [17].

Furthermore, Aleman et al. described four locations at which the electrodes were placed to detect SNAP: at the level of the infraorbital foramen, at the level of the maxillary foramen, at the level of the first spinal cord segment on midline, and at the level of the frontoparietal cerebral cortex [8]. These authors could not find a difference in the measurements of minimal SNCT at each location [8]. As we were looking for a fast and reliable examination in a clinical setting, we decided to examine only one representative location to speed up the process and reduce the risk of general anesthesia. The infraorbital foramen was elected for recording electrode placement, as this is easily accessible right under the skin surface. After gaining some experience, electrode placement and SNAP recording were uncomplicated and quick to perform.

In this study, the minimal SNCT of the infraorbital nerve was ≥ 15 mA in all healthy control horses with a mean of 18.75 mA (15–25 mA) compared to horses with i-TMHS, which demonstrated a mean minimal SNCT of 14.07 mA (5–25 mA). This finding supports the assumption of Aleman et al. (2013) that there is a functional impairment of the infraorbital nerve possibly causing a hypersensitivity in these horses [8]. However, not all horses with i-TMHS in our study population had a lower minimal SNCT ≤ 10 mA. This raises the question if branches of the trigeminal nerve other than the infraorbital nerve might be affected in these individuals, as Newton et al. (2007) proposed [7]. The authors suspected that the area of primary dysesthesia was frequently located in the caudal nasal cavity, as local anesthesia of the posterior ethmoidal nerve ameliorated the clinical signs of their study population [7]. Local anesthesia of the maxillary nerve in our study did not result in amelioration of HS clinical signs in most horses. This further supports the hypothesis, that dysfunction of the maxillary nerve is not the only cause of i-TMHS. Subsequently, physiologic minimal SNCT of the infraorbital nerve cannot be used to exclude i-TMHS as a diagnosis.

Minimal SNCT cannot be used to discriminate i-TMHS from s-TMHS. In two of the six horses with s-TMHS the minimal SNCT was pathologically decreased with ≤ 10 mA, comparable to i-TMHS. In horses with s-TMHS a lesion in an anatomic structure innervated by the trigeminal nerve or involving the trigeminal nerve itself can cause discomfort or pain [18]. Inflammatory lesions along the trigeminal nerve can lead to amplification of the perceived stimulus either via peripheral or in the central nervous system hyperexcitability [19]. Partially damaged and demyelinated neurons might trigger a burst of activity and recruit additional neighboring neurons, making them hyperexcitable since the myelin sheath is damaged and nerve fibers in close contact might additionally be depolarized [20]. In human medicine, removing this triggering lesion often causes relief of signs of trigeminal hyperesthesia [21].

Today, there is no consensual definition of which criteria must be fulfilled to diagnose i-TMHS, except that ‘other causes’ must be excluded. Therefore, Pickels et al. [1] proposed a diagnostic plan that involves signalment, patient history, observation of clinical signs, full clinical examination including ophthalmoscopy, ear endoscopy, examination of the oral cavity, endoscopy of the upper respiratory tract including guttural pouches, radiography of the head, CT, MRI and local anesthesia to rule out underlying causes of TMHS. We did not find amelioration of clinical signs after local anesthesia of the maxillary nerve in horses with i-TMHS as already described by others [7]. We therefore suggest the use of local anesthesia only to exclude a structural lesion as the cause of headshaking.

Aleman et al. showed that, in one horse with seasonal occurrence of clinical signs of headshaking, which was measured off season, SNCT was higher than in i-TMHS horses that were symptomatic at the time measured, but lower than in healthy horses [8]. Therefore, a reversibility in functional impairment of the nerve was discussed [8]. Furthermore, Aleman et al. raised the question of whether clinical sign severity or seasonality correlates with SNCT [8]. In our study, one of four horses, which had a Talbot score of 0, and only recently had stopped shaking, showed a decreased minimal SNCT ≤ 10 mA, whereas the other three had a minimal SNCT at 15 mA and 20 mA. We therefore assume that, in some cases, functional changes may take some time to reverse while being off season, or that, in chronic cases, neuropathic changes are not reversed despite the temporary absence of clinical signs.

In summary, SNCT measurements at the infraorbital nerve are quick and easy to perform; nevertheless general anesthesia is needed. SNAP are reliably acquired at this location and are repeatable. A decrease in SNCT confirms the diagnosis of neuropathy causing TMHS. Nevertheless a normal SNCT does not rule out TMHS, considering the present understanding of this disease. Unfortunately SNCT measurements do not differentiate s-TMHS from i-TMHS. If this method is used, it is considered prudent that individual reference values are established, as differences may exist between certain devices and the examiner’s methods.

### Electronic supplementary material

Below is the link to the electronic supplementary material.

Supplementary Material 1. Supplemental table 1 shows signalment, diagnosis and underlying structural pathology and performed local anesthesia (if applicable) of healthy control horses and horses with signs of headshaking. Empty cells indicated missing data because data were not documented (signalment), information is not applicable (structural diagnosis), local anesthesia was not performed, or measurement could not be performed due to technical problems (SNCV measurements; e.g. artifacts). One horse (number 44) was excluded, because no SNAP could be recorded due to technical problems sensory nerve conduction stimulus threshold = SNCT; milli Ampere = mA; nervus = N; sensory nerve action potential = SNAP; micro Volts = µV; meter per second = m/s; male = m; female = f; neutered = n, positive= +; negative=-.

Supplementary Material 2

## Data Availability

The datasets supporting the conclusions of this article are included within the article and its additional file (supplemental data).
