# Supplementary material for: Sensory nerve conduction stimulus threshold measurements of the infraorbital nerve and its applicability as a diagnostic tool in horses with trigeminal-mediated headshaking
Source: BMC Vet Res. 2024 May 16;20:201. doi: 10.1186/s12917-024-04068-x (PMC11097574; doi:10.1186/s12917-024-04068-x)
Supplement: Supplementary file 1 — Supplementary Material 1. Supplemental table 1 shows signalment, diagnosis and underlying structural pathology and performed local anesthesia (if applicable) of healthy control horses and horses with signs of headshaking. Empty cells indicated missing data because data were not documented (signalment), information is not applicable (structural diagnosis), local anesthesia was not performed, or measurement could not be performed due to technical problems (SNCV measurements; e.g. artifacts). One horse (number 44) was excluded, because no SNAP could be recorded due to technical problems sensory nerve conduction stimulus threshold = SNCT; milli Ampere = mA; nervus = N; sensory nerve action potential = SNAP; micro Volts = µV; meter per second = m/s; male = m; female = f; neutered = n, positive= +; negative=-. [file 12917_2024_4068_MOESM1_ESM.docx]

|  |  | **right infraorbital nerve** | | | **left infraorbital nerve** | | |
| --- | --- | --- | --- | --- | --- | --- | --- |
|  |  | **minimal SNCT**  **(mA)** | **amplitude**  **(µV)** | **SNCV**  **(m/s)** | **minimal SNCT**  **(mA)** | **amplitude**  **(µV)** | **SNCV**  **(m/s)** |
| **horse 1** | concentric | 15 | 42 | 68 | 25 | 23 | 79 |
|  | monopolar | 15 | 21 | 85 | not measurable | |  |
| **horse 2** | concentric | not performed | |  | 20 | 19 | 79 |
|  | monopolar | not performed | |  | 20 | 14 | 106 |
| **horse 3** | concentric | 20 | 124 | 69 | 20 | 25 | 73 |
|  | monopolar | not measurable | |  | 15 | 63 | 75 |
| **horse 4** | concentric | 20 | 10 | 79 | 15 |  | 57 |
|  | monopolar | not measurable | |  | not measurable | |  |
| **horse 5** | concentric | 20 | 44 | 73 | not measurable | |  |
|  | monopolar | 15 | 99 | 73 | not measurable | |  |

**Supplemental Table 1:** Comparison of sensory nerve conduction measurements recorded with two monopolar needle electrodes and with one bipolar concentric needle electrode in unaffected horses. With monopolar needle electrodes, sensory nerve conduction potentials were regularly not measurable due to artifacts.

SNCT = sensory nerve conduction stimulus threshold; SNCV = sensory nerve conduction velocity; mA= milliamperes; µV= microvolt; m/s = meter/second; concentric = recording with one bipolar concentric needle electrode; monopolar = recording with two monopolar needle electrodes.
